# Supplementary material for: Impact of anionic lipids on the energy landscape of conformational transition in anion exchanger 1 (AE1)
Source: Nat Commun. 2025 Nov 26;16:11664. doi: 10.1038/s41467-025-66786-6 (PMC12748791; doi:10.1038/s41467-025-66786-6)
Supplement: Supplementary file 1 — Supplementary Information [file 41467_2025_66786_MOESM1_ESM.pdf]

Supporting Information

Supplementary Figures

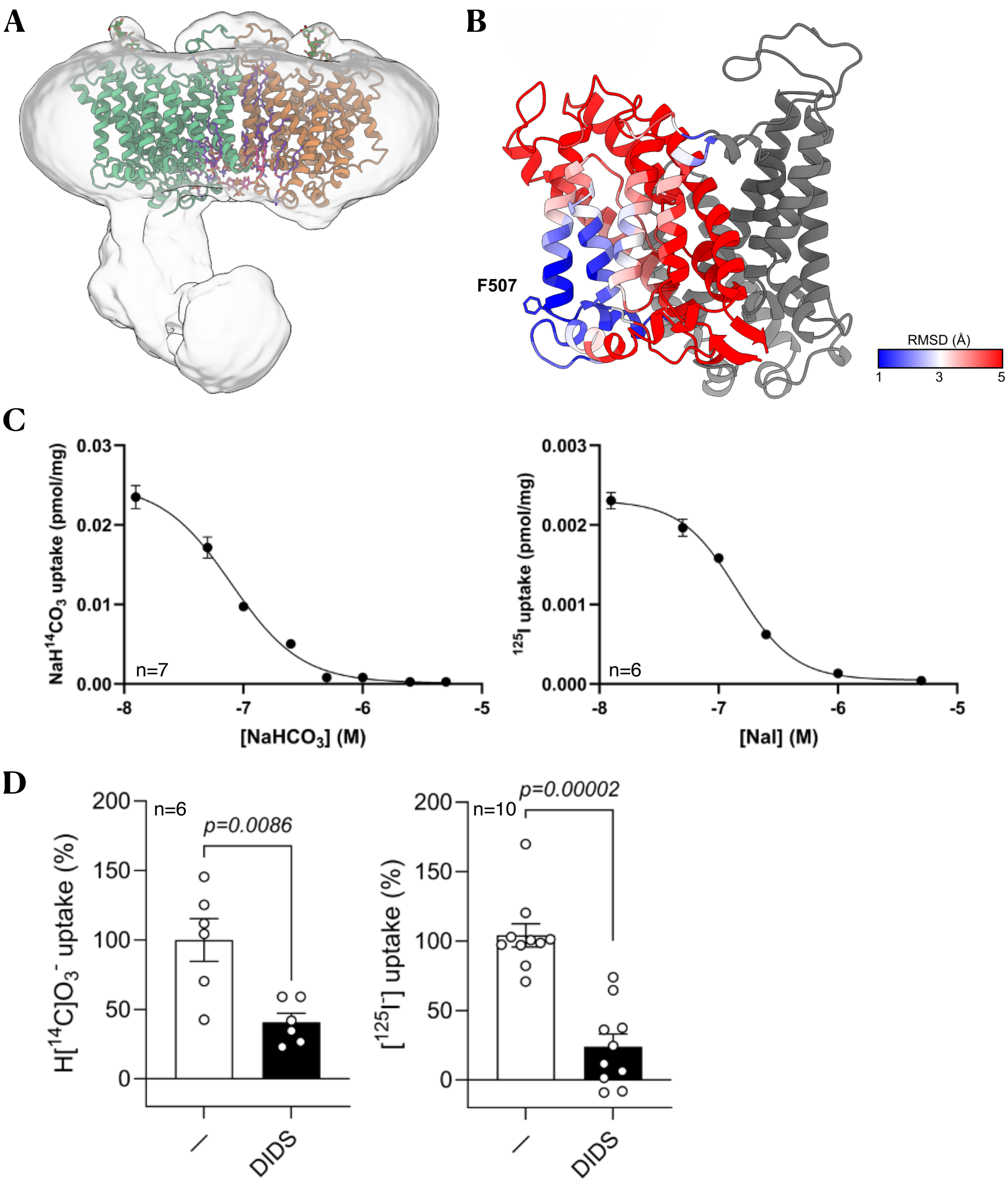

Figure S1: **cryo-EM density and substrate uptake by AE1** (A) AE1 IF1-OF model (IF1 in orange and OF in green) superimposed with the low-threshold map highlights the low-resolution density of the cytosolic domain. (B) Structural alignment between AE1 protomers in IF1 and OF state (colored by RMSD), shows that the movement of the TD doesn't affect the residue F507. (C) AE1-specific 10-s uptake of 10 µM $NaH[^{14}C]O_3$ or $Na[^{125}I]$ was measured with an isotopic dilution of $NaHCO_3$ or NaI, yielding a $\log(EC_{50}) = -7.10 \pm 0.02$ for $NaHCO_3$ (corresponding to a mean of 79 nM) and $\log(EC_{50}) = -6.84 \pm 0.01$ for NaI (corresponding to a mean of 143 nM). Data are mean ± SD of ≥ 6 biological replicates as indicated in the panel. (D) 1-min uptake of 10 µM $NaH[^{14}C]O_3$ or $Na[^{125}I]$ into proteoliposomes containing AE1 was measured in the presence or absence of 1 mM DIDS. Data are presented as mean ± SEM (n ≥ 6 as indicated in the panels). The distribution of the biological replicates was assessed with the Shapiro–Wilk test. For statistical comparisons, a two-tailed Mann–Whitney U test was applied to non-parametric data, and a two-tailed unpaired Student's t-test was used for parametric data. $\log(EC_{50})$ values were obtained by fitting the data to a nonlinear regression model using GraphPad Prism 10.

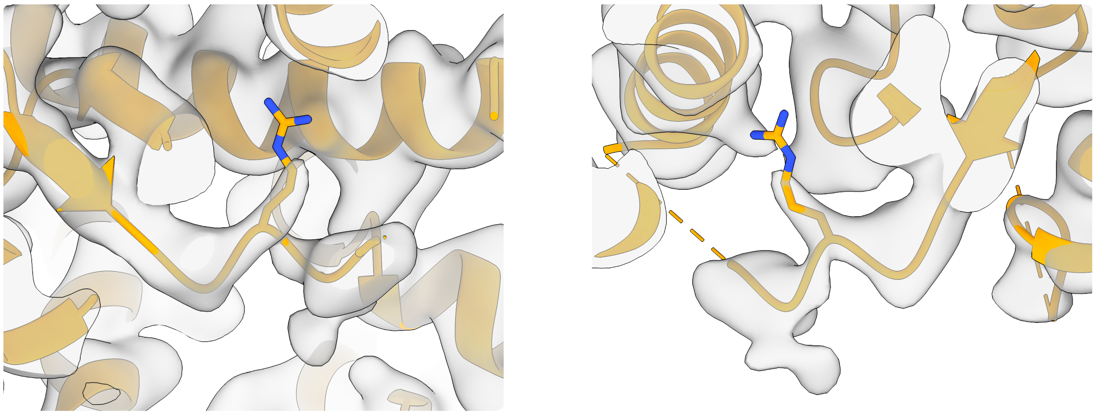

Figure S2: **Close-up view of the model-to-map fit for TM10 in the IF1 protomer from the AE1 OF–IF1 structure.** Left and right panels show different views of the cryo-EM density and the fitted atomic model for TM10 and residue R730. A Gaussian filter (σ = 1.2) and a threshold level of 0.1 were applied in UCSF Chimera.

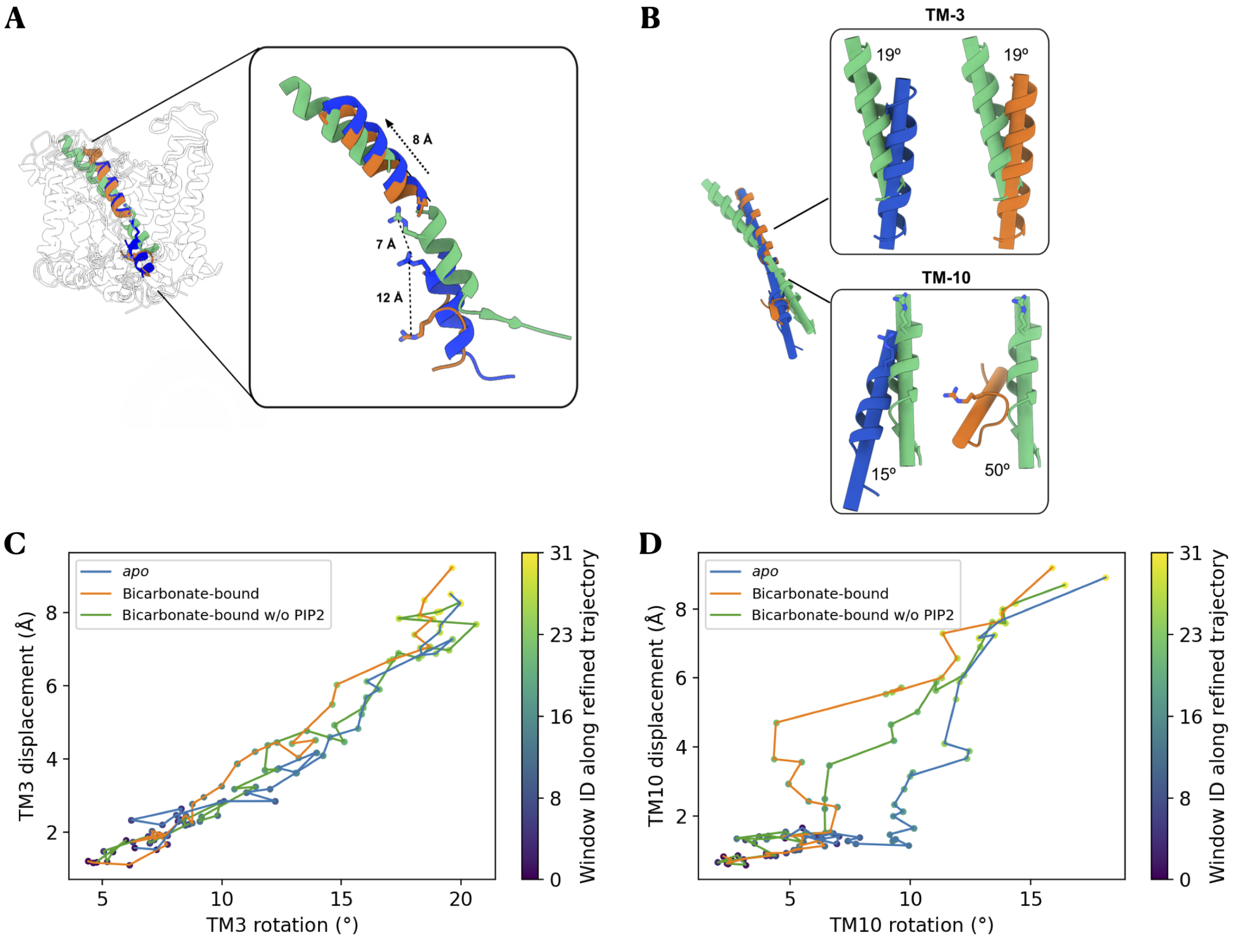

Figure S3: **TM3 and TM10 rotation and displacement.** (A) TM3 shifts by 8 Å between the IF (blue and orange) and OF (green) states, while R730 moves 7 Å between the OF and IF2 (blue) states and 12 Å between the IF2 and IF1 (orange) states. (B) Comparison of the rotation angles between TM3 and TM10 among the three conformational states, with colors matching those shown in A. (C) Calculated rotation and displacement of TM3 along SMwST trajectories for the three simulation systems: *apo*, HCO_3^--bound, and HCO_3^--bound without PIP_2. Points represent the averaged center for each window with color from dark blue to yellow indicating windows 0 to 31. (D) Calculated rotation and displacement of TM10 along SMwST trajectories.

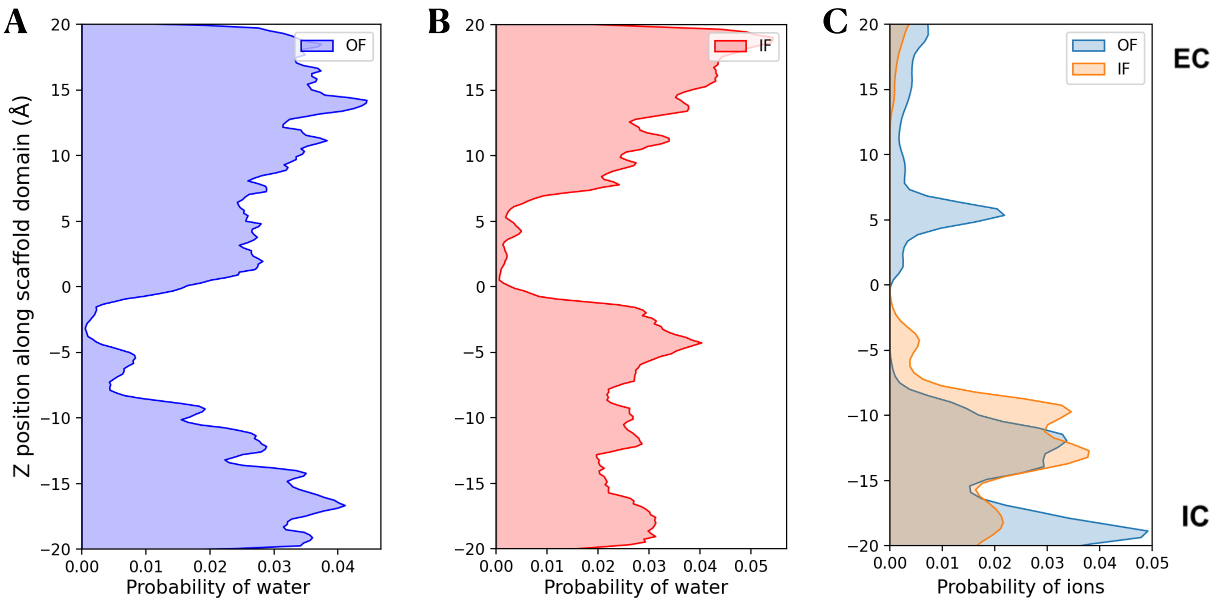

Figure S4: **Water and ion density in the AE1 lumen.** (A) Hydration probability of the lumen along the z-axis in the OF state during the equilibrium simulation. (B) Hydration probability in the IF state. (C) Anion distribution along the z-axis in both OF and IF states aligned using the SD, with the negative side representing the cytoplasm.

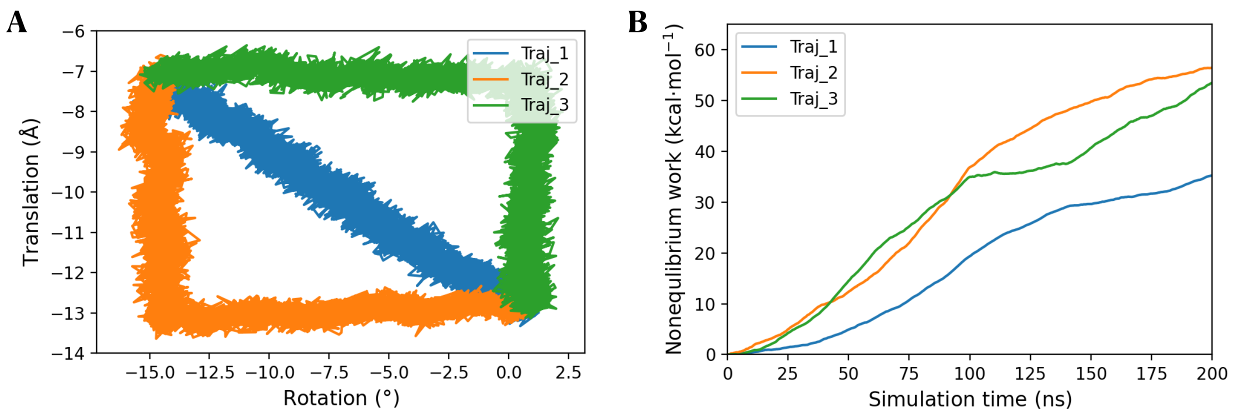

Figure S5: **Different pulling protocols and associated non-equilibrium work.** (A) Three different pulling protocols were tested for transition from OF to IF, using the same simulation times and force constants: (1) first rotating and then translating (green), (2) pulling in the reverse order (orange), and, (3) pulling synchronously (blue). (B) The non-equilibrium work generated from the three pulling protocols.

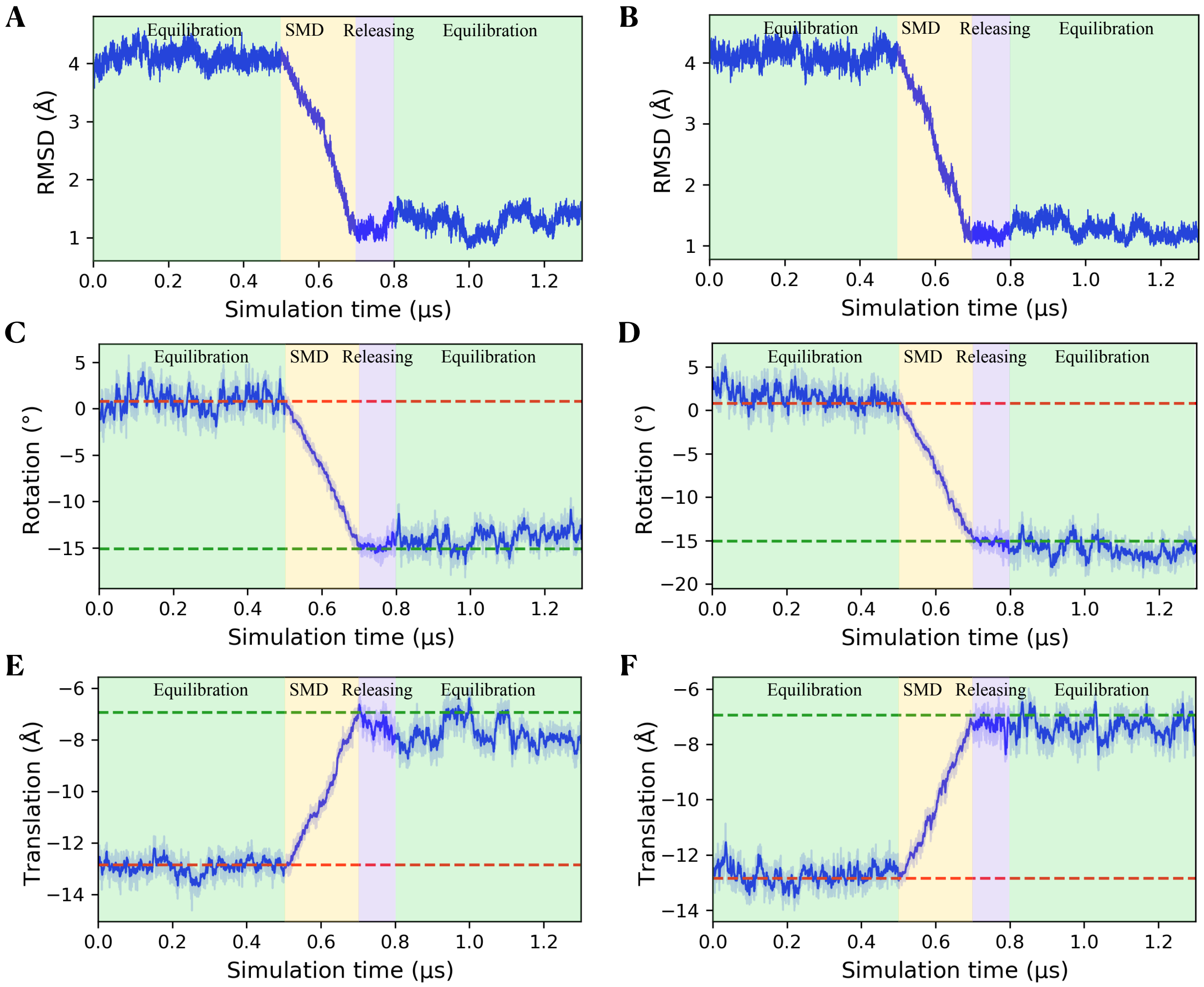

Figure S6: **RMSD and CV changes during the transition simulations of the *apo* and the substrate-bound PIP_2-removed AE1 systems.** (A and B) Cα RMSD of TM helices of the TD during the driven simulation with respect to the target structure in the IF state of the *apo* (A) and the substrate-bound, PIP_2-removed (B) systems. (C and D) The TD rotation CV changes during the equilibration, driven, releasing, and final free equilibration stages for the *apo* (C) and the PIP_2-removed (D) systems. (E and F) The TD translation CV changes of the *apo* (E) and the PIP_2-removed (F) systems.

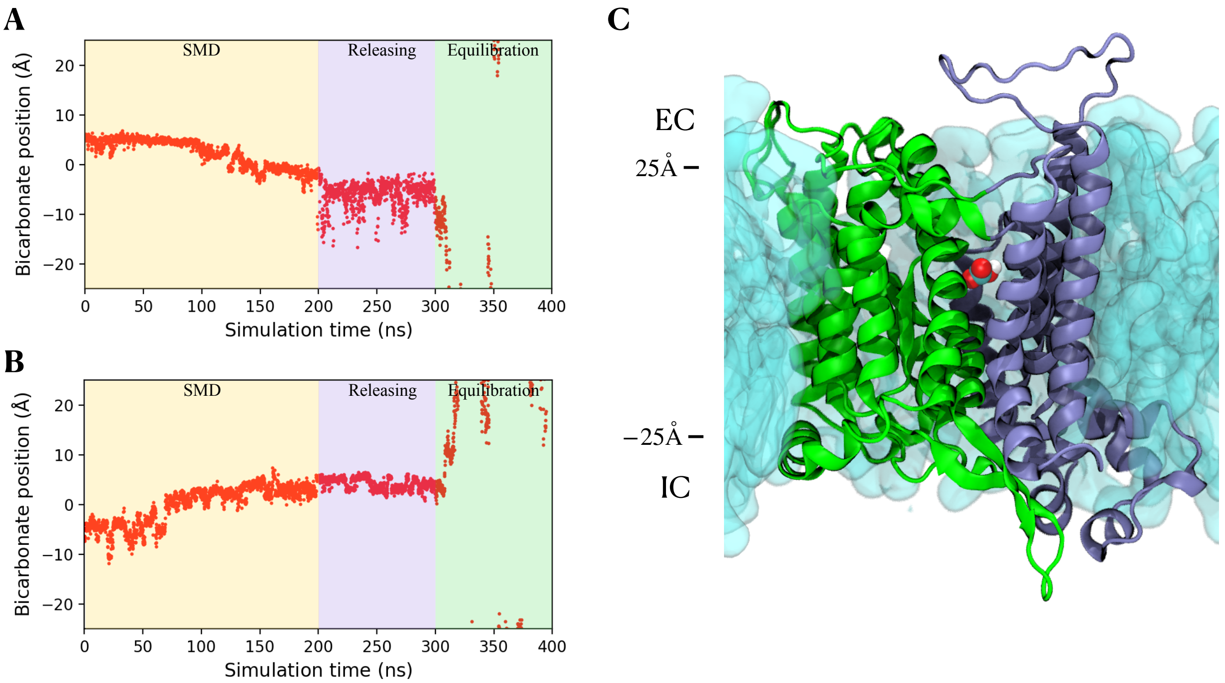

Figure S7: **HCO_3^- transport in AE1 during state transition.** (A) z coordinate of HCO_3^- during the state transition from OF to IF2, releasing, and final free equilibration stages. The frames are aligned using the SD. (B) z coordinate of HCO_3^- during the reverse process (IF2 to OF transition). (C) Transmembrane spanning of AE1 (numbers matching those in Panel B. The membrane spans from −25 Å to 25 Å. The bound HCO_3^- is located at z = 5 Å in the OF state.

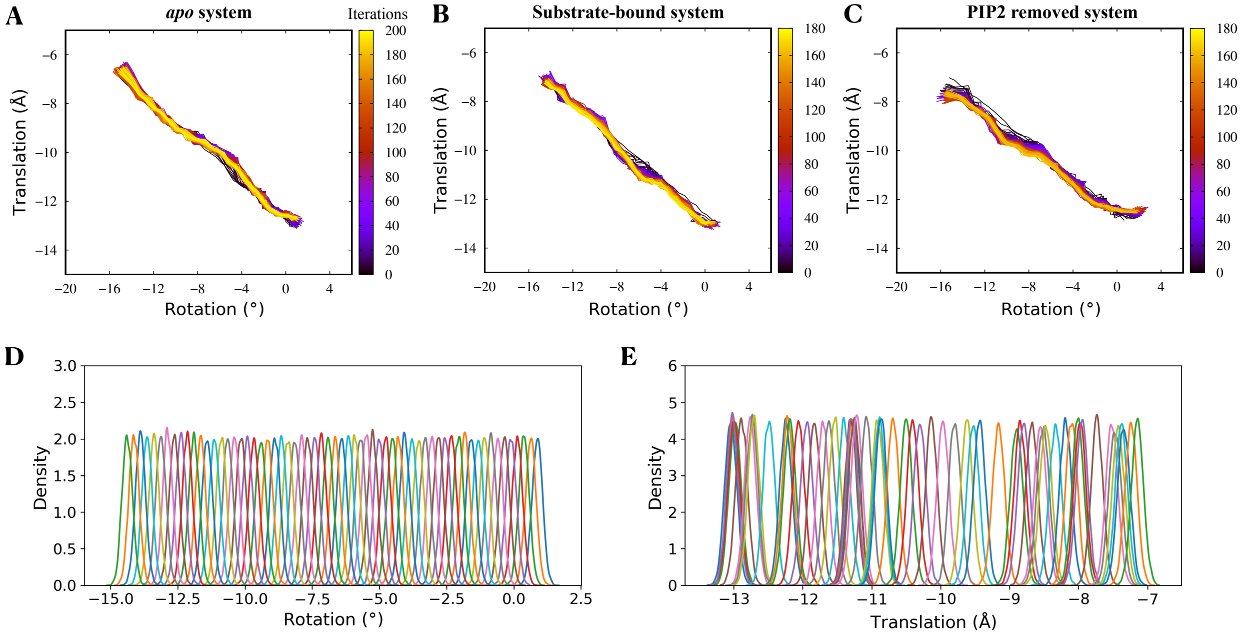

Figure S8: **Convergence of enhanced sampling simulations.** (A) The refinement of the transition pathway during 200 iterations of SMwST simulations for the *apo* system. The color is changing from black to yellow to show the iterations. (B) The pathway refinement for the substrate-bound system. (C) The pathway refinement for the substrate-bound, PIP_2-removed system. (D) The histograms for the rotation CV from BEUS simulations. The density curve of each window is calculated using the Guassian KDE function. (E) The histograms for the translation CV.

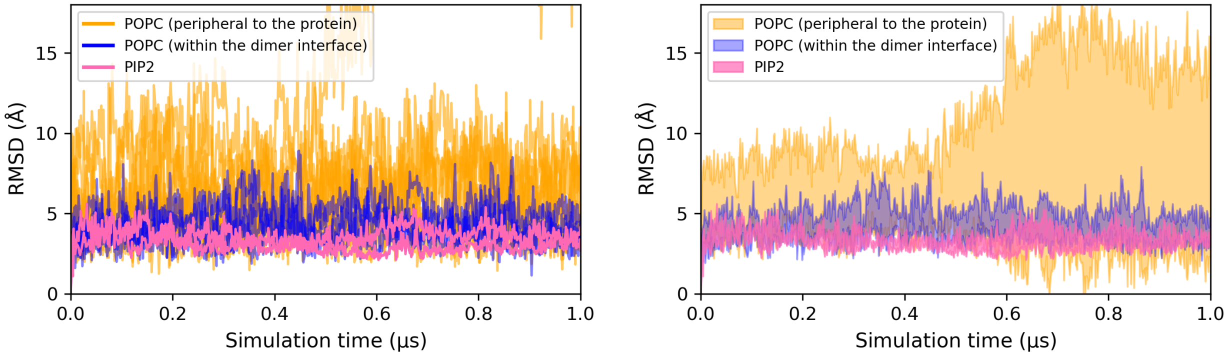

Figure S9: **RMSD of cryo-EM resolved lipids in MD.** (A) Headgroup RMSD during the 1 μs equilibrium simulation for the three groups of lipids relative to their initial positions are shown: 8 peripheral POPC lipids (orange), 4 POPC lipids within the dimer interface (blue), and 2 PIP_2 lipids (pink). (B) Standard deviation of the RMSD values for the same group, indicating the variability over the simulation time.

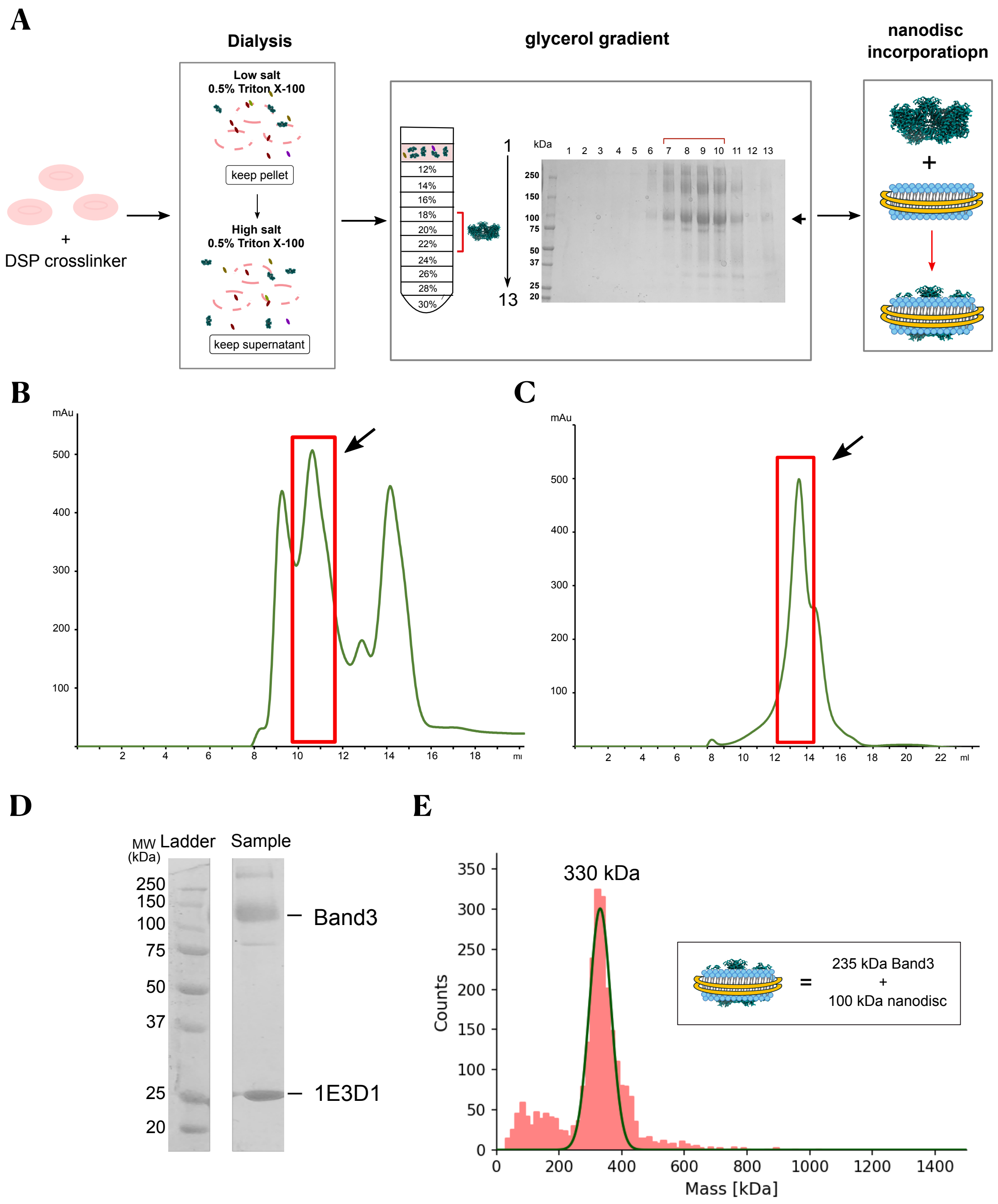

Figure S10: **Purification and biochemical characterization of human AE1.** (A) Human erythrocyte ghost membranes, represented as pink empty erythrocytes, were washed and solubilized using 0.5% (w/v) Triton-X100. Membrane fragments were then added in a 12-30% glycerol gradient. Fractions collected were loaded in an SDS-PAGE gel. Gradient fractions containing AE1 are marked with a red line. AE1 was incorporated in MSP nanodiscs. (B) Size exclusion chromatography of the fractions containing AE1 in nanodisc using a Superdex 200 column. The second peak (marked with the red box) was collected. (C) Collected fractions were subjected to a second round of size exclusion chromatography using a Superose 6 column. (D) SDS-PAGE gel of fraction that corresponds to the main peak from the gel filtration in panel C. (E) Mass photometry analysis of the size exclusion chromatography peak from the second gel filtration. The peak corresponds to a population of particles with a size around 330 kDa that represents the AE1 dimer in MSP nanodisc.

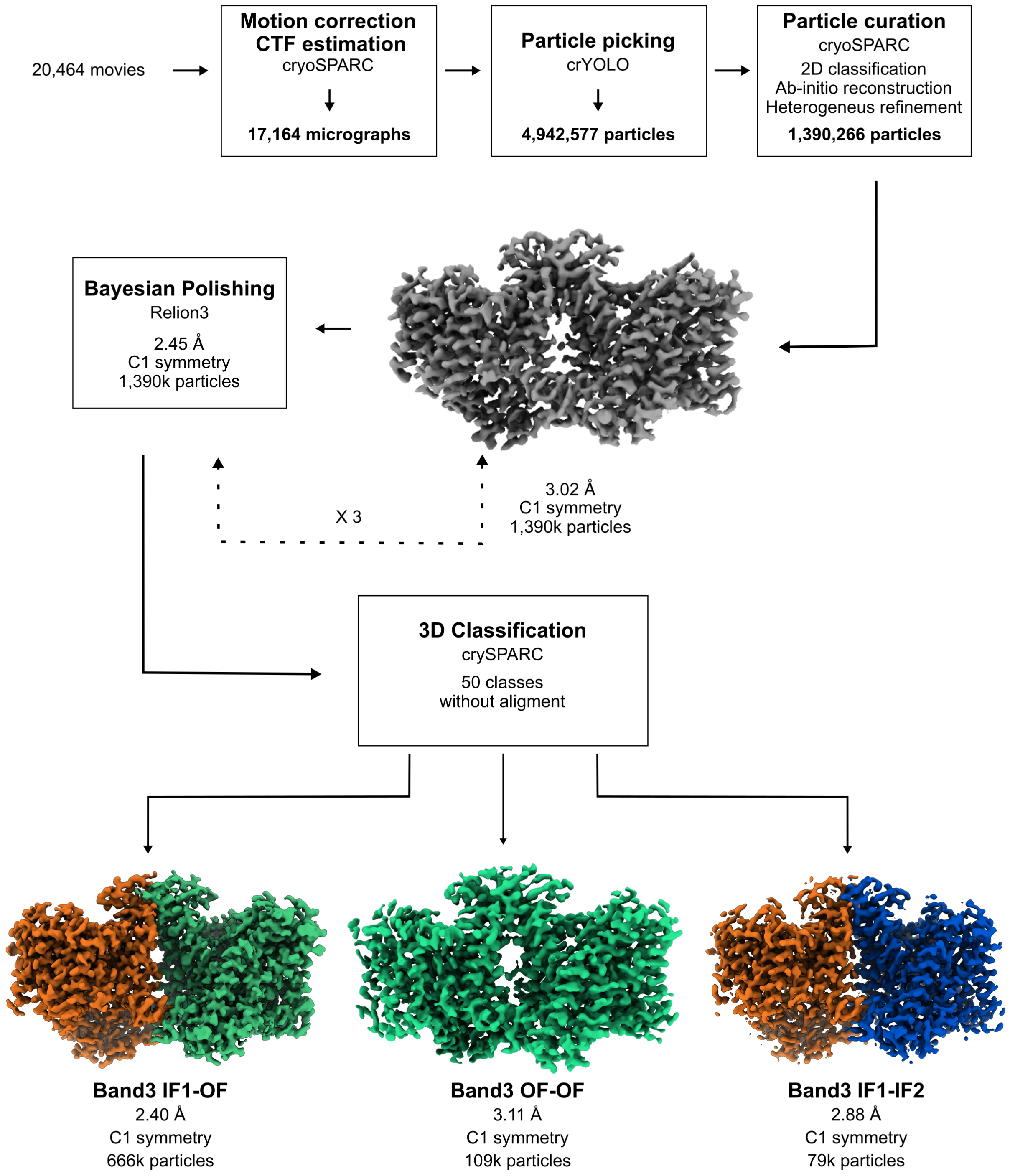

Figure S11: **Cryo-EM workflow.** Flowchart outlining cryo-EM image acquisition and processing performed to obtain the structure of AE1 in three different conformations. All processing was performed using CryoSPARC v.3.2 and Relion 4 (see Methods for details).

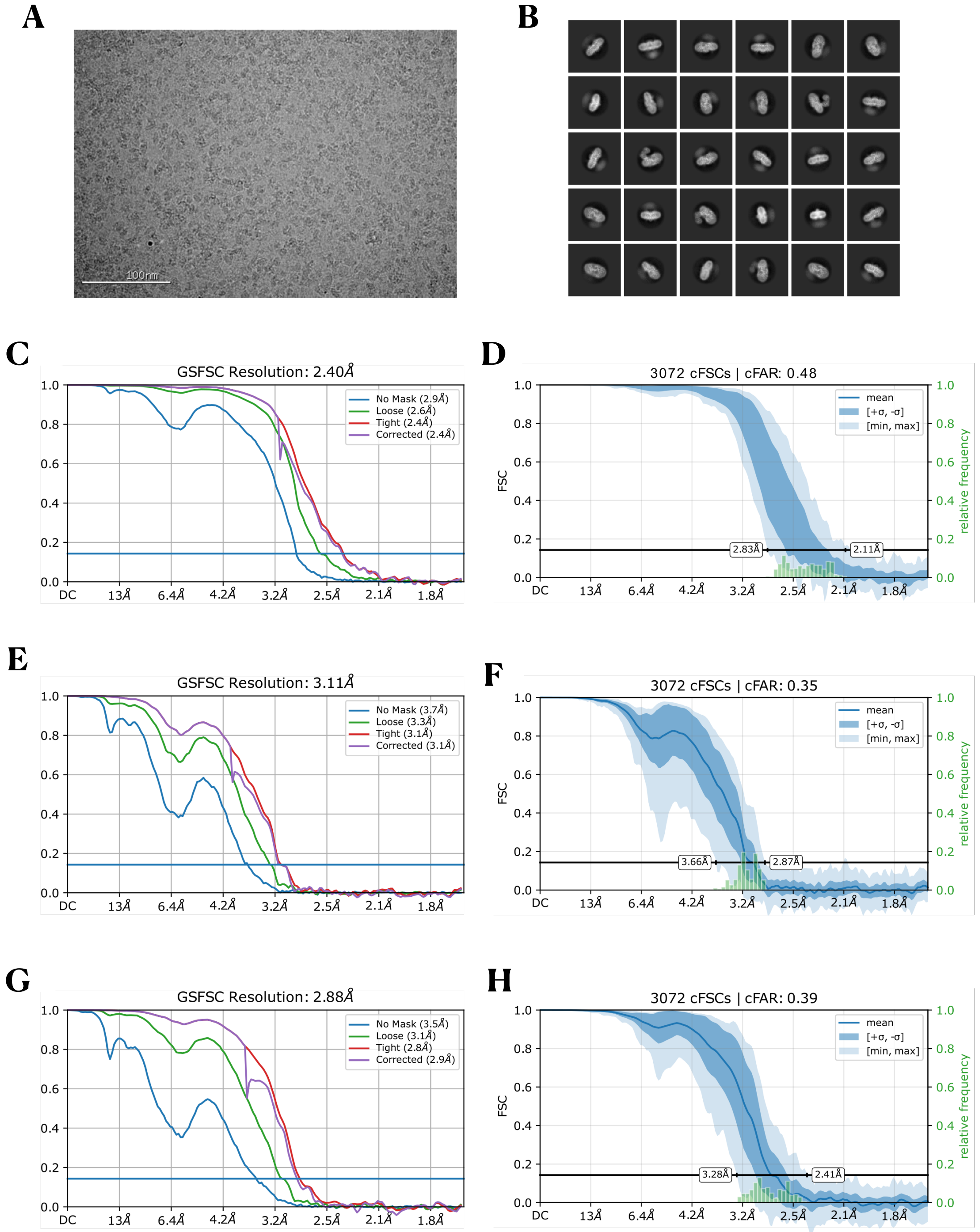

Figure S12: **Single particle cryo-EM structure determination.** (A) A representative micrograph. (B) 2D class averages of particles initially picked with crYOLO (~1.3M),[52] ordered by population. (C) Fourier shell correlation (FSC) curves for the AE1 dimer in the IF1-OF structure. (D) Directional anisotropy of the IF1-OF structure, as calculated by Orientation Diagnostics in CryoSPARC 4.5. (E) FSC curves for the AE1 dimer in OF-OF structure. (F) Directional anisotropy of the OF-OF structure, as calculated by Orientation Diagnostics in CryoSPARC 4.5. (G) FSC curves for the AE1 dimer in IF1-IF2 structure. (H) Directional anisotropy of the IF1-IF2 structure, as calculated by Orientation Diagnostics in CryoSPARC 4.5.

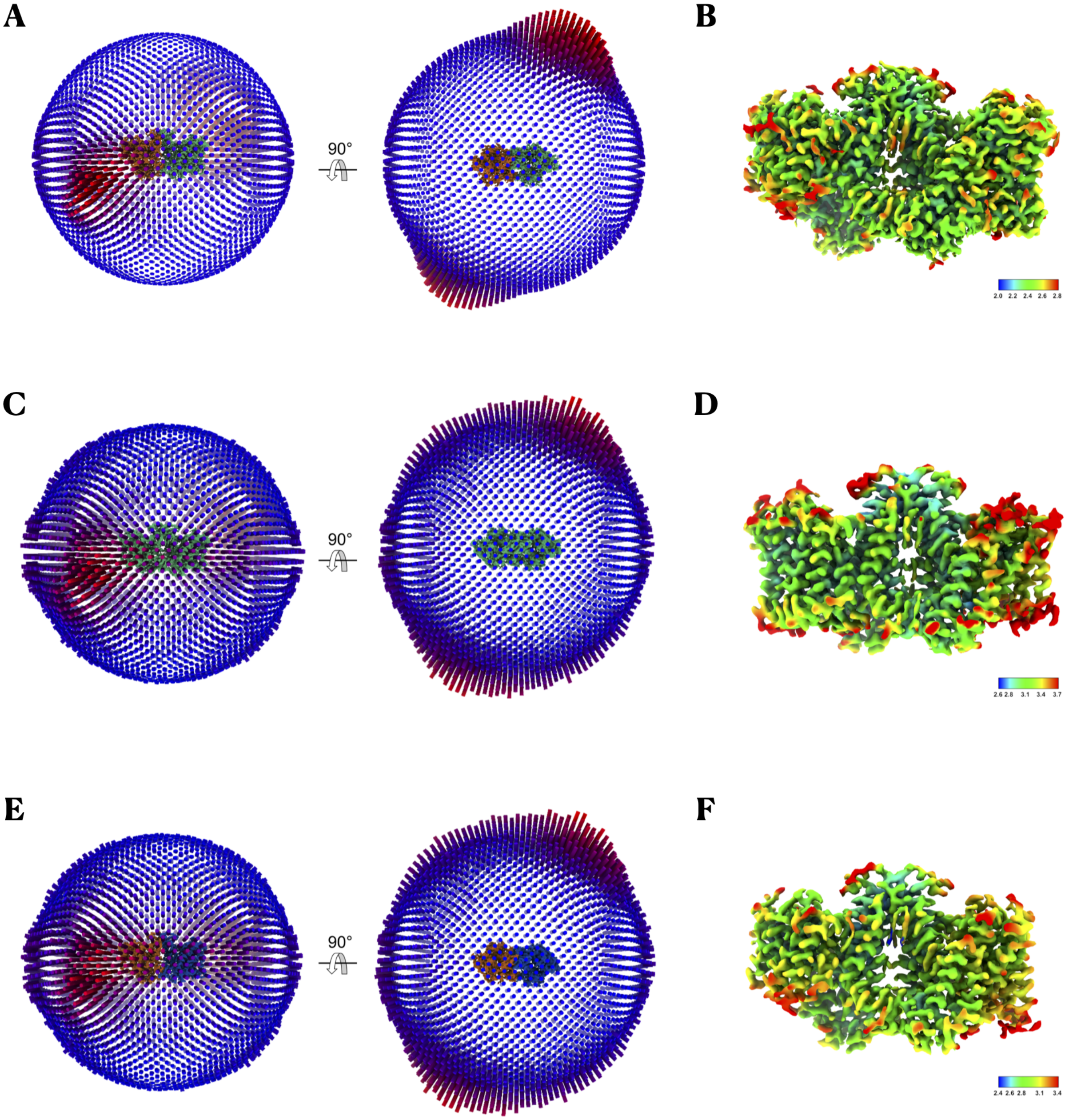

Figure S13: **Cryo-EM map plots.** (A) Orientation distribution plot for the 666,254 particles used in the IF1-OF refinement. (B) Local resolution plot of the cryo-EM map from the global 3D reconstruction for AE1 IF1-OF structure. (C) Orientation distribution plot for the 108,734 particles used in the OF-OF refinement. (D) Local resolution plot of the cryo-EM map from the global 3D reconstruction for AE1 OF-OF structure. (E) Orientation distribution plot for the 78,596 particles used in the AE1 IF1-IF2 refinement. (F) Local resolution plot of the cryo-EM map from the global 3D reconstruction for AE1 IF1-IF2 structure.

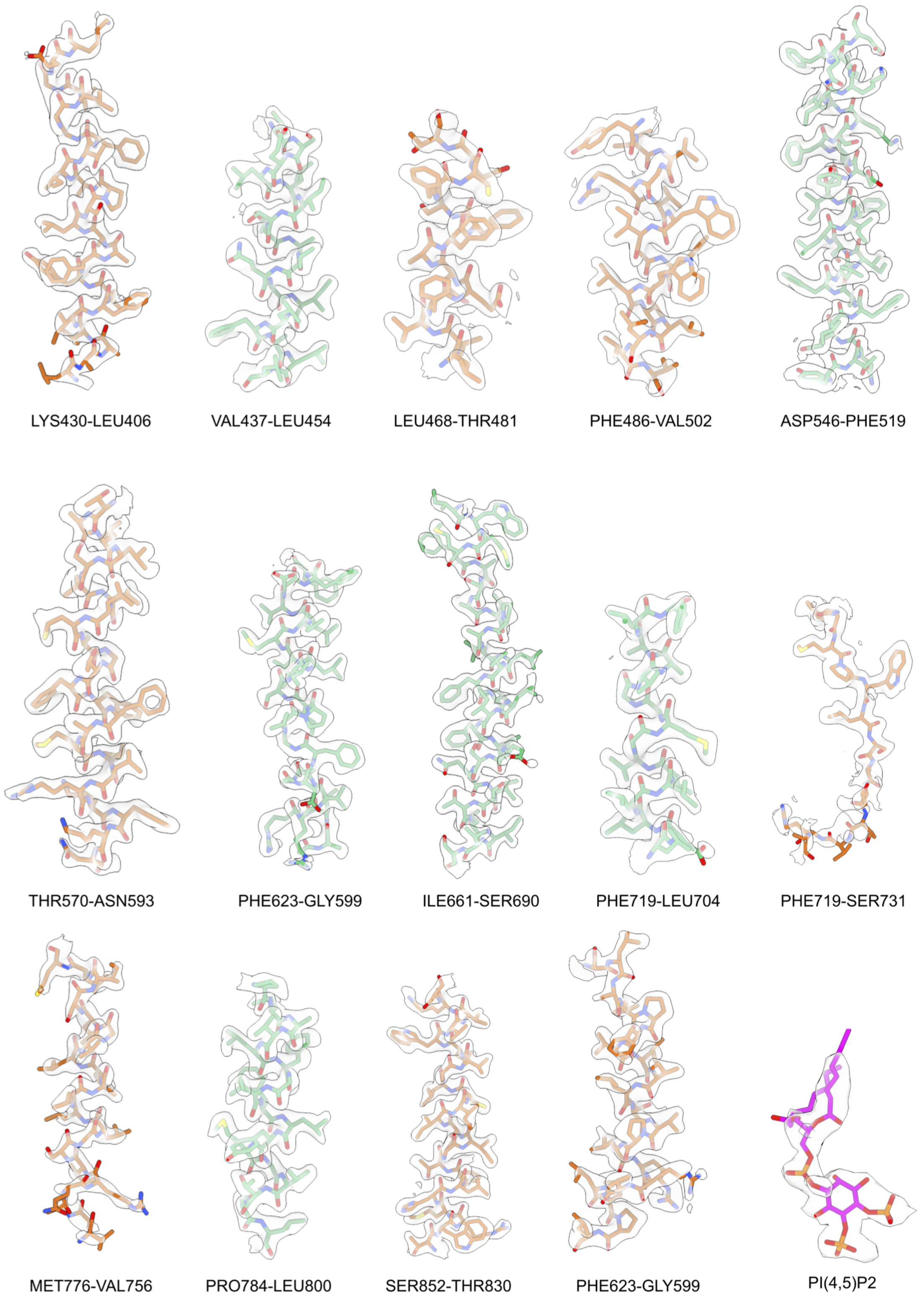

Figure S14: **Model/map fit.** Cryo-EM densities (transparent gray surface) are shown with corresponding segments of the AE1 IF1-OF atomic model; side chains are rendered in stick representation and colored as in Fig. 1.

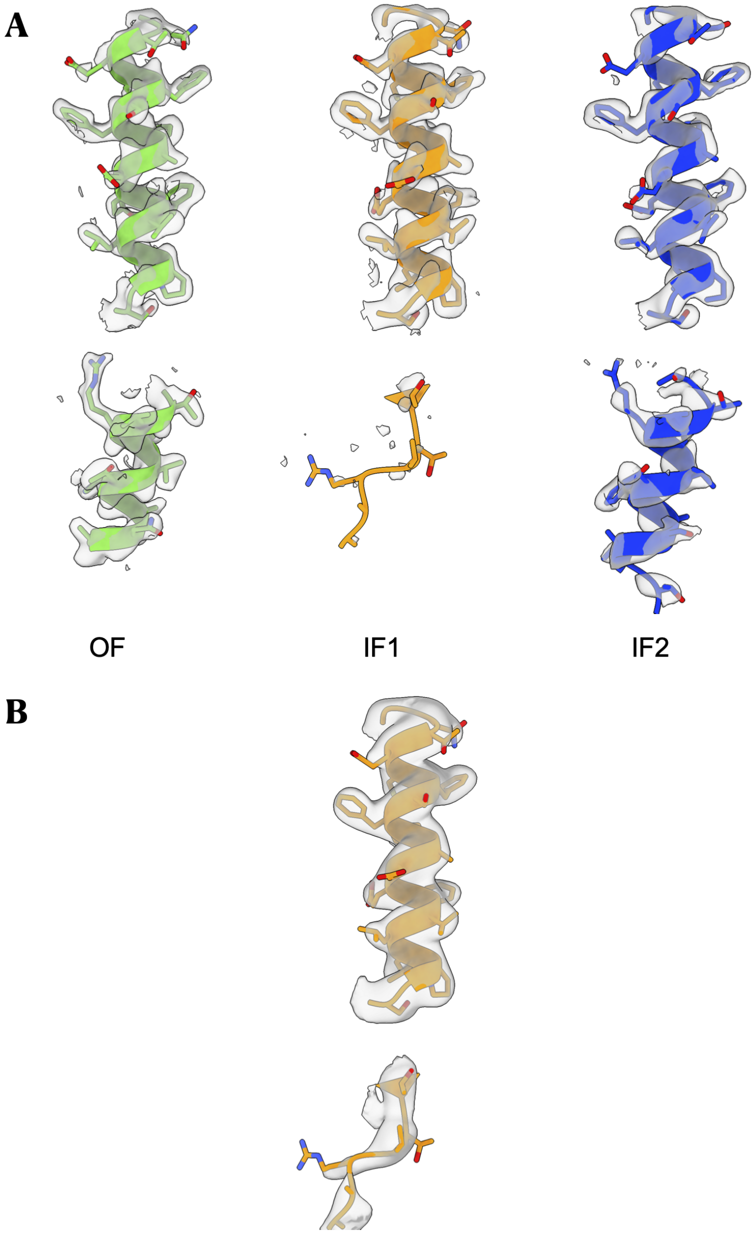

Figure S15: **Map-to-model fits for TM3 and TM10 across three states.** (A) Model fits and corresponding cryo-EM density maps are shown for TM3 (residues 465–483) and TM10 (residues 727–739, or 727–732 in IF1) from the OF, IF1, and IF2 states of AE1. (B) Map-to-model fit of IF1 helices with a low-pass filtering.

Supplementary Tables

	AE1 IF_1-OF<br>PDB 9MND<br>EMD-48421	AE1 OF-OF<br>PDB 9MNG<br>EMD-48422	AE1 IF_1-IF_2<br>PDB 9MOS<br>EMD-48480
**Data collection and processing**			
Magnification	105,000x	105,000x	105,000x
Voltage (kV)	300	300	300
Electron exposure (e^-/Å^2)	58	58	58
Defocus range (μm)	-0.5 to -1.5	-0.5 to -1.5	-0.5 to -1.5
Pixel size (Å)	0.83	0.83	0.83
Symmetry imposed	C1	C1	C1
Initial micrographs	20,087	20,087	20,087
Final micrographs	17,164	17,164	17,164
Initial particle images	1,781,329	1,781,329	1,781,329
Final particle images	666,254	108,734	78,596
Map resolution (Å)	2.4	3.11	2.88
FSC threshold	0.143	0.143	0.143
**Model composition**			
Non-hydrogen atoms	8519	8310	8240
Protein residues	982	1034	971
Ligand molecules	26	8	18
**RMS Deviations**			
Bond lengths (Å)	0.008	0.002	0.005
Bond angles (°)	1.222	0.493	0.885
**Validation**			
Clashscore	2.77	4.5	5.53
Poor rotamers (%)	2.42	2.93	0.97
**Ramachandran plot**			
Favored (%)	97.22	98.34	97.18
Allowed (%)	2.57	1.81	2.82
Disallowed (%)	0.21	0	0
**EMRinger Score**	3.89	4.44	3.55

Table S1: **Cryo-EM data collection, refinement and validation statistics**

**System information**	
Simulation box dimensions	145×145×125 Å^3
Total number of atoms	276k
Total number of water molecules	59k
**Salt Concentration (mM)**	
Bicarbonate	18
Chloride	87
Potassium (neutralize)	165
**Lipid Composition**	
POPC	143
PSM	105
POPE	121
cholesterol	316
POPS	60
PIP_2	6
PIP_3	6

Table S2: **MD simulation system setup**
