# Supplementary material for: Impact of anionic lipids on the energy landscape of conformational transition in anion exchanger 1 (AE1)
Source: Nat Commun. 2025 Nov 26;16:11664. doi: 10.1038/s41467-025-66786-6 (PMC12748791; doi:10.1038/s41467-025-66786-6)
Supplement: Supplementary file 2 — Description of Additional Supplementary Files [file 41467_2025_66786_MOESM2_ESM.pdf]

Description of Additional Supplementary Files

File Name: Supplementary Movie 1

Description: Conformational changes, bicarbonate transport, and PIP2 interactions during the OF-IF transition in AE1. In this movie, an extracellular bicarbonate initially diffuses and binds to R730 of an AE1 protomer in the OF state. The protein then undergoes the OF to IF transition under the applied biases. Concurrently, the salt bridge between PIP2 and K743 is disrupted. Finally, bicarbonate dissociates from the binding site and is translocated to the cytoplasmic side.
