# Supplementary figures and images for: Impact of anionic lipids on the energy landscape of conformational transition in anion exchanger 1 (AE1)
Source: Nat Commun. 2025 Nov 26;16:11664. doi: 10.1038/s41467-025-66786-6 (PMC12748791; doi:10.1038/s41467-025-66786-6)

## Slide 1
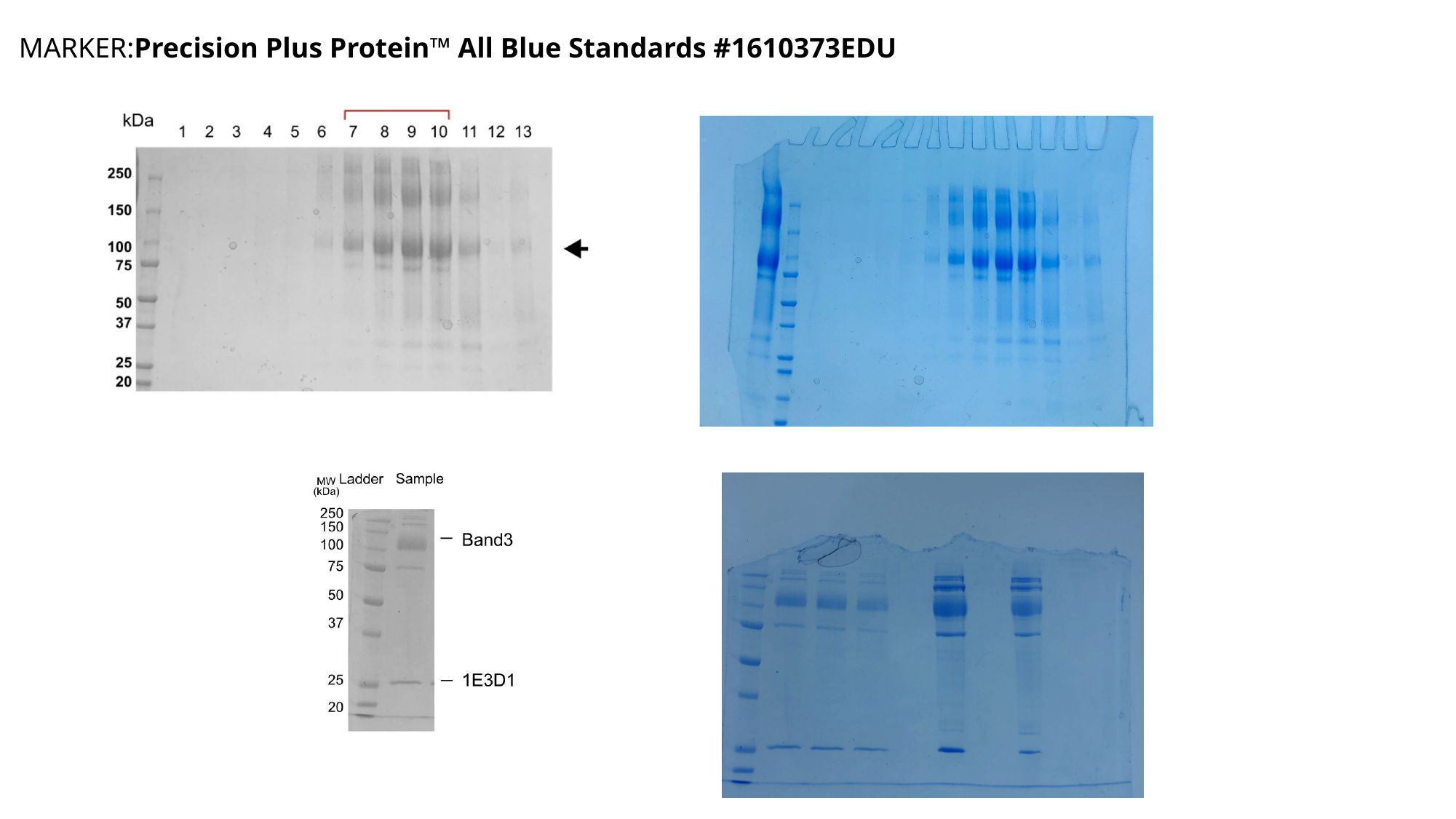

MARKER:Precision Plus Protein™ All Blue Standards #1610373EDU

Supplement: Supplementary file 6 — Source Data [file 41467_2025_66786_MOESM6_ESM.zip › SourceData/Supplementary_fig_10d_original_gel.pptx]
